# SARS-CoV-2 potential drugs, drug targets, and biomarkers: a viral-host interaction network-based analysis

**DOI:** 10.1038/s41598-022-15898-w

**Published:** 2022-07-13

**Authors:** Asmaa Samy, Mohamed A. Maher, Nehal Adel Abdelsalam, Eman Badr

**Affiliations:** 1 University of Science and Technology, Zewail City, Giza 12578 Egypt; 2 Faculty of Pharmacy, Cairo University, Cairo, 11562 Egypt; 3 Faculty of Computers and Artificial Intelligence, Cairo University, Giza, 12613 Egypt

**Keywords:** Diagnostic markers, Computational models, Data integration, Gene regulatory networks

## Abstract

COVID-19 is a global pandemic impacting the daily living of millions. As variants of the virus evolve, a complete comprehension of the disease and drug targets becomes a decisive duty. The Omicron variant, for example, has a notably high transmission rate verified in 155 countries. We performed integrative transcriptomic and network analyses to identify drug targets and diagnostic biomarkers and repurpose FDA-approved drugs for SARS-CoV-2. Upon the enrichment of 464 differentially expressed genes, pathways regulating the host cell cycle were significant. Regulatory and interaction networks featured hsa-mir-93-5p and hsa-mir-17-5p as blood biomarkers while hsa-mir-15b-5p as an antiviral agent. MYB, RRM2, ERG, CENPF, CIT, and TOP2A are potential drug targets for treatment. HMOX1 is suggested as a prognostic biomarker. Enhancing HMOX1 expression by neem plant extract might be a therapeutic alternative. We constructed a drug-gene network for FDA-approved drugs to be repurposed against the infection. The key drugs retrieved were members of anthracyclines, mitotic inhibitors, anti-tumor antibiotics, and CDK1 inhibitors. Additionally, hydroxyquinone and digitoxin are potent TOP2A inhibitors. Hydroxyurea, cytarabine, gemcitabine, sotalol, and amiodarone can also be redirected against COVID-19. The analysis enforced the repositioning of fluorouracil and doxorubicin, especially that they have multiple drug targets, hence less probability of resistance.

## Introduction

Owing to its unpredicted prognosis and disease course, COVID-19 is considered one of the most peculiar challenges facing the human race. In less than three years, SARS-CoV-2 infected more than 300 million people globally and caused death to more than 5 million^1^. It began in December 2019 when a novel coronavirus (CoV) was identified in Wuhan, China^2^. It belongs to the betacoronavirus genus that includes severe acute respiratory syndrome coronavirus (SARS-CoV) and Middle East respiratory syndrome coronavirus (MERS-CoV)^3^. Accordingly, the International Committee on Taxonomy of Viruses (ICTV) announced the official name of the virus as severe acute respiratory syndrome coronavirus-2 (SARS-CoV-2) and the disease it causes as COVID-19^4^. Furthermore, due to the rapid human-to-human transmission, the World Health Organization (WHO) declared COVID-19 as a global public health emergency in January 2020 and a pandemic in March the same year^5^. Lately, the WHO declared the Omicron variant a variant of concern due to mutations in the viral genome that may affect the virus spread^6^. It has been verified in 155 different locations around the globe^7^.

Although SARS-CoV-2 infection causes similar symptoms to SARS-CoV and MERS-CoV, the pathophysiology of COVID-19 and the molecular basis of the disease severity are not fully understood yet. Recent studies highlighted the role of host immunity and inflammation in disease progression. Sudden storms of cytokines and chemokines may lead to lung injury and acute respiratory distress syndrome (ARDS), which is considered the clinical hallmark of SARS-CoV-2 infection. It has also been shown that viral replication results in cellular apoptosis that may cause diffuse alveolar damage, leading to ARDS as well^8,9^. Cellular apoptosis can be attributed to the fact that viruses lack the needed machinery for replication. Therefore, they rely entirely on the host cells to express the required viral proteins for this process. To efficiently control host cell resources, viruses may either inhibit or induce cell cycle progression. With such interference, some viral infections may eventually cause cancer. Previous studies confirmed that many CoVs induce host cell cycle arrest at different phases, such as G0/G1 by SARS-CoV^10^, porcine epidemic diarrhea virus^11^, and murine hepatitis virus^12^. Bronchitis virus^13^ and transmissible gastroenteritis virus^14^ induce cell cycle arrest at S and G2/M phases. Understanding the cellular pathways associated with viral infection can reveal potential targets for therapy.

One therapeutic approach that has been widely investigated to fight the pandemic is the microRNA (miRNA)-based approach^15^. miRNAs are small non-coding RNA molecules of ~21 nucleotides that regulate gene expression at post-transcriptional level^16^. They are involved in the regulation of different cellular pathways, including viral infection and host cell response^17^. A recent review by Abedi et al.^15^ summarized the role of miRNAs in SARS-CoV-2 life cycle and pathogenesis. For instance, hsa-mir-125a-5p and hsa-mir-200 families target the angiotensin-converting enzyme 2 and consequently block viral attachment and entry^18^. hsa-let-7e-5p^18^ and hsa-mir-98-5p^19^ target transmembrane protease, serine 2 (TMPRSS2). Other miRNAs have been reported to inhibit viral replication and protein synthesis. Additionally, hsa-miR-323a-5p and hsa-mir-20b-5p were predicted to have an antiviral effect by reducing inflammatory responses and preventing lung injury^20^. Besides being potential antiviral agents, miRNA might be diagnostic biomarkers as reported by a recent study that the disease severity and mortality in aged patients may have resulted from the lower expression levels of host miRNAs^21^.

**Figure 1:**
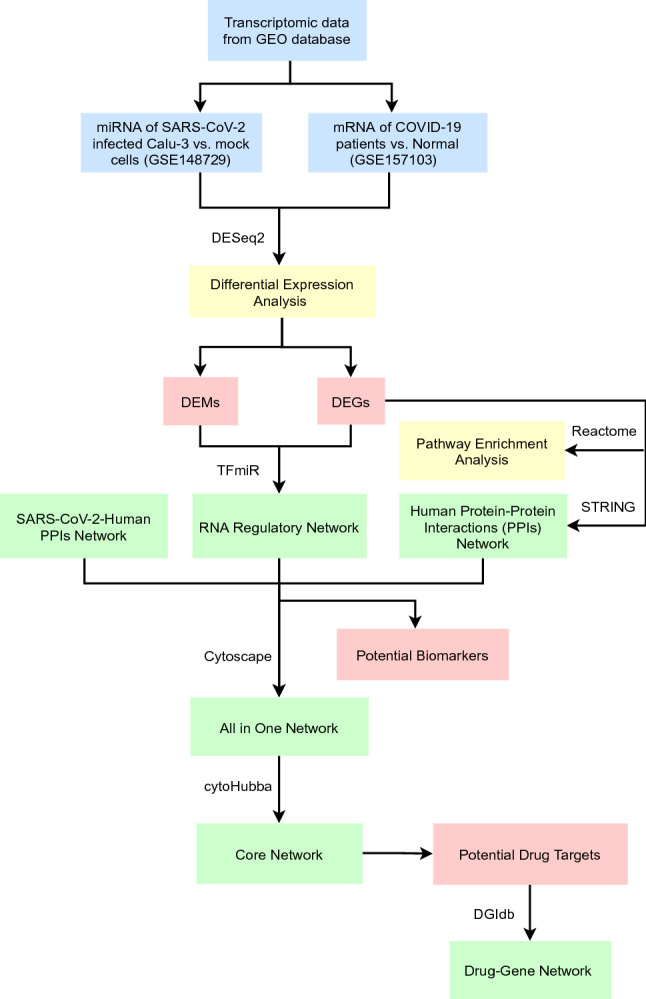
A block diagram depicting the workflow for identifying potential biomarkers and drug targets for COVID-19 infection.

As SARS-CoV-2 is an ongoing health hazard with multiple variants emerging in a short time interval, accelerated research is urgently needed to develop prevention, diagnosis, and treatment strategies against the pandemic. The development of new antiviral agents is a challenging, costly long-term process, especially with the rapid evolvement of viral variants. Likewise, resistance adds another layer of complexity to the discovery and design of antivirals^22^. Drug repurposing or repositioning is a promising strategy in this situation to save both time and cost amidst a pandemic^23^. In this study, we applied an integrated computational pipeline of transcriptomic profiling and network analysis, as shown in Fig. 1. Our analysis aimed to (i) enhance the understanding of the molecular mechanism behind SARS-CoV-2 pathogenesis, (ii) identify candidate key targets and their repurposed FDA-approved drugs, and (iii) identify candidate biomarkers for the disease diagnosis. Hence, our study proposes MYB, TOP2A, RRM2, ERG, CENPF, CIT, and HMOX1 as potential drug targets. Moreover, hsa-mir-93-5p and hsa-mir-17-5p are two miRNAs that have been identified as potential biomarkers for COVID-19 infection. hsa-mir-15b-5p is a promising natural miRNA-based antiviral agent. Anthracyclines, mitotic inhibitors, anti-tumor antibiotics, and CDK1 inhibitors are key classes for repositioning against coronavirus infection. Fluorouracil and doxorubicin are two FDA-approved anticancer drugs and have multiple target genes. They could be repurposed to treat COVID-19 upon thorough experimentation and validation.

## Results

### Differential expression analysis and biological insights

Differential expression analysis was performed on GSE157103^66^ and GSE148729^67^ to identify differentially expressed genes (DEGs) in plasma and leukocyte samples and differentially expressed microRNAs (DEMs) in lung cancer cell lines, respectively. The dataset GSE157103 holds expression profiles for blood samples from patients tested positive for SARS-CoV-2 and other tested negative. From GSE148729 dataset, only small RNA expression profiles were retrieved for analysis. These samples were SARS-CoV-2 infected and non-infected Calu-3 human epithelial cell line.

**Figure 2:**
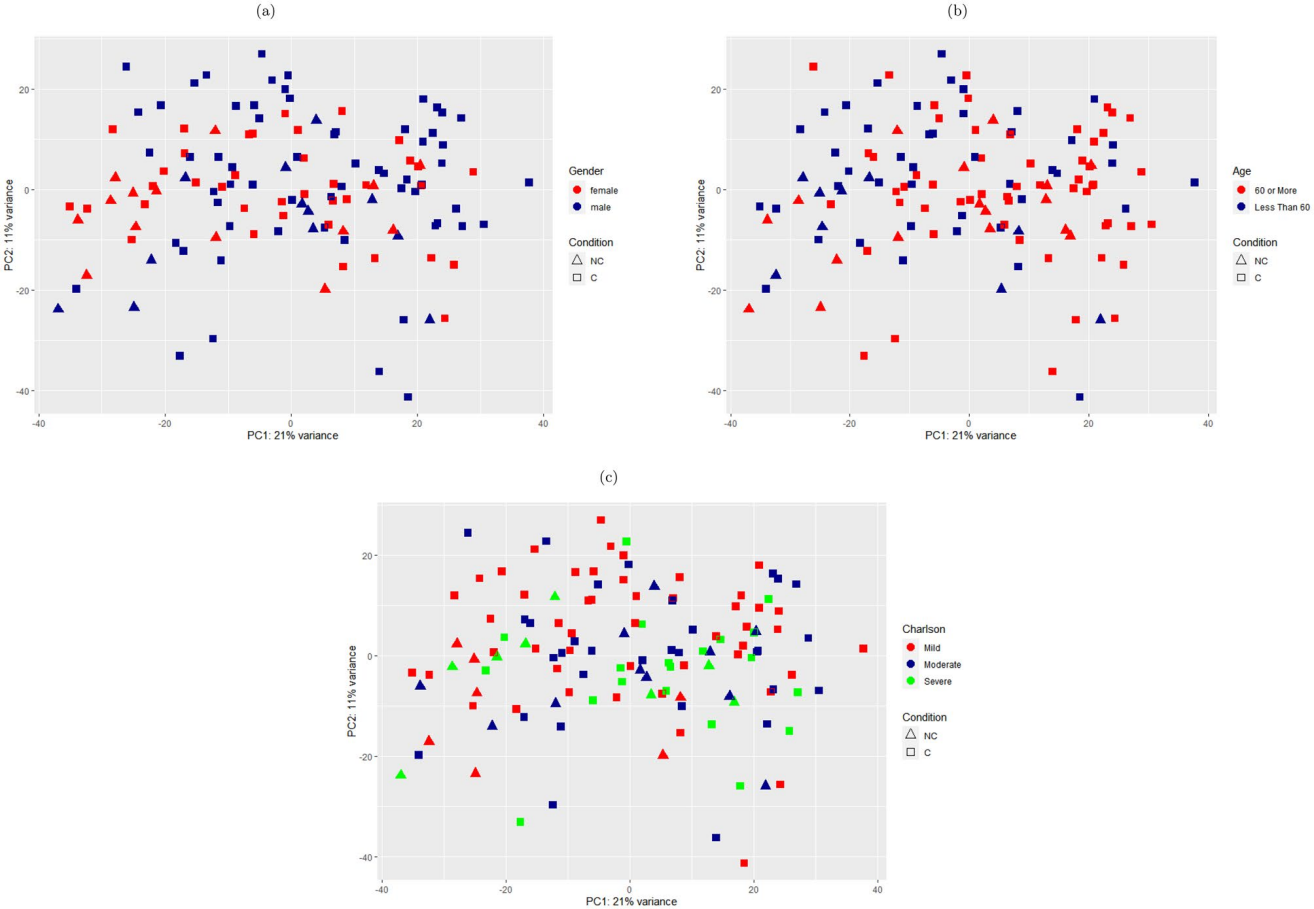
PCA plots showing that (**a**) gender, (**b**) age, and (**c**) Charlson score have no confounding effects on gene expression between COVID-19 patients and non-COVID-19 samples (GSE157103).

For GSE157103, we utilized the Principal Component Analysis (PCA) plots for gender, age, and Charlson severity score to ensure that those factors are not sources of variation. For age, data were split into two groups, below 60 years and 60 years or above. The age groups had 53 samples below 60 years and 73 with 60 years or above. The Charlson score was categorized into three groups: below 3 for mild, between 3 and 6 is moderate, and 6 or more for severe cases. The groups had 56, 42, and 28 samples, respectively. The samples were split into 74 males and 52 females. As Fig. 2 demonstrates, there was no defined separation or clustering between the two cohorts based on the abovementioned characteristics.

**Figure 3:**
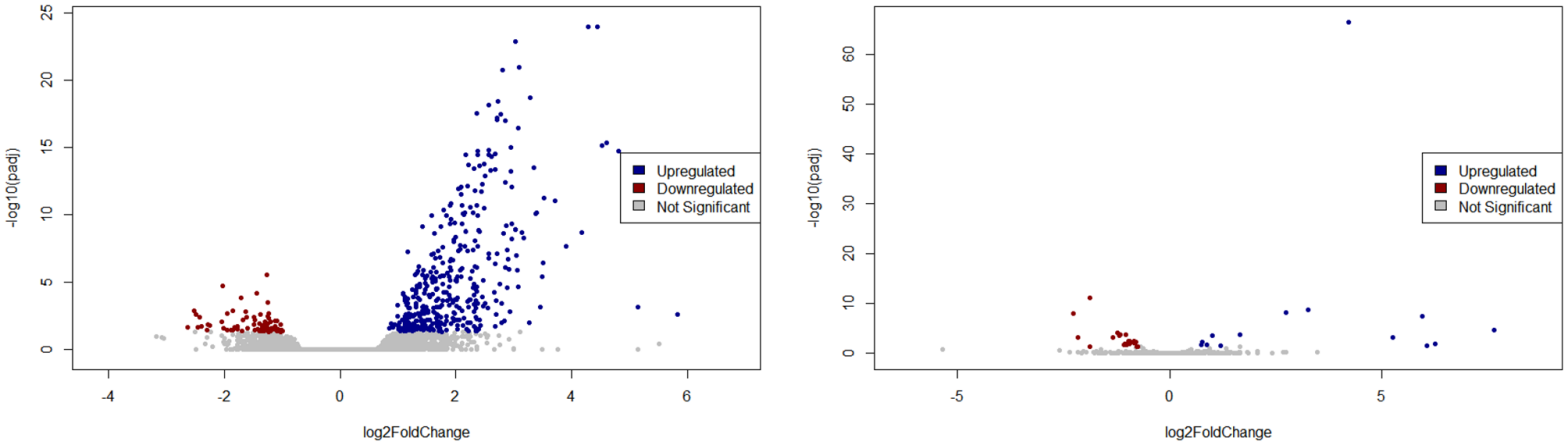
Volcano plots of (**a**) differentially expressed genes and (**b**) differentially expressed miRNAs by analyzing GSE157103 and GSE148729 datasets, respectively. Blue circles: upregulated DEGs/DEMs; red circles: downregulated DEGs/DEMs; grey circles: non-significantly expressed mRNAs/miRNAs.

Based on statistical thresholds of adjusted *P*-value < 0.05 and $|Log_2foldchange~(FC)| > 0.5$, we identified 464 DEGs (389 upregulated and 75 downregulated genes) from GSE157103 dataset (Fig. 3 and Supplementary Table 1). We also identified 37 DEMs (15 upregulated and 22 downregulated miRNAs) from GSE148729 dataset (Fig. 3 and Supplementary Table 2). Same statistical thresholds were set for DEMs and DEGs.

Reactome pathway analysis results showed that the DEGs of patients with COVID-19 were significantly enriched in 97 pathways with maximum False Discovery Rate (FDR) of 0.01 (Supplementary Table 3). The top listed pathways with lowest FDR values were cell cycle, including different phases of mitotic cell cycle and cell cycle checkpoints, DNA replication, and RHO GTPase effectors for signaling as given in Table 1.

**Table 1:** Top 20 enriched pathway terms of DEGs in SARS-CoV-2 patients with lowest FDR values.

Pathway name	Entities *P* value	Entities FDR
G1/S transition	1.11E−16	1.75E−14
G1/S-specific transcription	1.11E−16	1.75E−14
Cell cycle, mitotic	1.11E−16	1.75E−14
Cell cycle	1.11E−16	1.75E−14
Mitotic G1 phase and G1/S transition	1.11E−16	1.75E−14
Cell cycle checkpoints	1.11E−16	1.75E−14
Polo-like kinase mediated events	4.04E−14	5.46E−12
Amplification of signal from unattached kinetochores via a MAD2 inhibitory signal	1.03E−13	1.08E−11
Amplification of signal from the kinetochores	1.03E−13	1.08E−11
Resolution of sister chromatid cohesion	1.77E−13	1.69E−11
Mitotic spindle checkpoint	5.63E−13	4.84E−11
Mitotic prometaphase	4.85E−12	3.63E−10
G2/M checkpoints	4.97E−12	3.63E−10
EML4 and NUDC in mitotic spindle formation	2.20E−11	1.47E−09
G0 and Early G1	4.48E−11	2.82E−09
M phase	8.15E−11	4.81E−09
RHO GTPase effectors	2.16E−10	1.19E−08
RHO GTPases activate formins	3.14E−10	1.63E−08
DNA replication	3.90E−10	1.95E−08
Activation of ATR in response to replication stress	6.63E−10	3.12E−08

### Identification of biomarkers and drug targets

**Figure 4:**
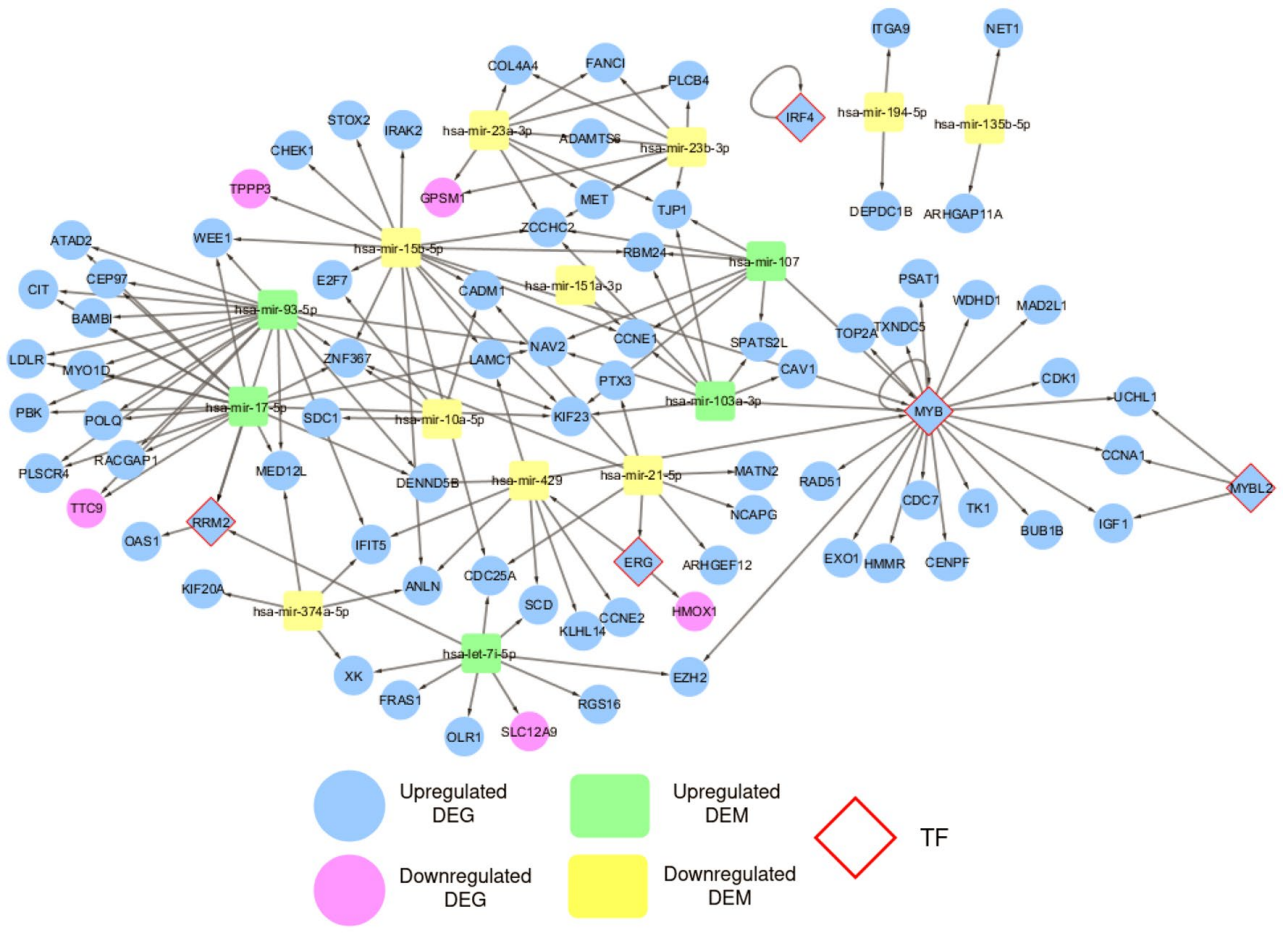
RNA regulatory network that consists of 96 nodes and 150 edges. Node’s color and shape are mapped to node type as labeled in the figure. The network is a directed graph to indicate the interaction direction.

Different interactions were retrieved and studied for a wide-scope analysis of the DEGs and DEMs. First, RNA regulatory network was constructed to identify interactions between DEGs and DEMs. The network consists of 96 nodes, including 81 DEGs (5 Transcription Factors (TFs); RRM2, ERG, MYB, MYBL2, and IRF4) and 15 DEMs. It has 150 edges, representing different types of interactions; one TF-miRNA, 24 TF-gene, 117 miRNA-gene, and 8 miRNA-TF (Fig. 4 and Supplementary Table 4). Among the five transcription factors, MYB had the highest interaction by regulating transcription of 17 genes and being regulated by 4 miRNAs. The network revealed 4 upregulated DEMs (hsa-mir-93-5p, hsa-mir-17-5p, hsa-mir-103a-3p, and hsa-mir-107) that could be considered as potential biomarkers for the disease diagnosis.

Superimposing virus-human interactome network on the regulatory network highlighted three common nodes. The two upregulated DEGs, CENPF and CIT, interact with SARS-CoV-2 nsp13. Moreover, HMOX1 is a downregulated DEG that interacts with SARS-CoV-2 ORF3a. On the regulatory level, CENPF is regulated by MYB-TF, CIT is regulated by two upregulated DEMs (hsa-mir-93-5p and hsa-mir-17-5p), and HMOX1 is regulated by ERG-TF.

Merging the two previous networks resulted into 452 nodes (of which 410 are human proteins). Physical and functional interactions of 275 human proteins were retrieved from STRING (1, 062 queries) and overlaid onto the merged network, resulted into the All in One network consists of 452 nodes connected by 1, 544 edges represent three types of interactions: regulatory interactions among miRNAs-TFs-genes, virus-host Protein-Protein Interactions (PPIs), and human PPIs (Supplementary Table 5).

**Figure 5:**
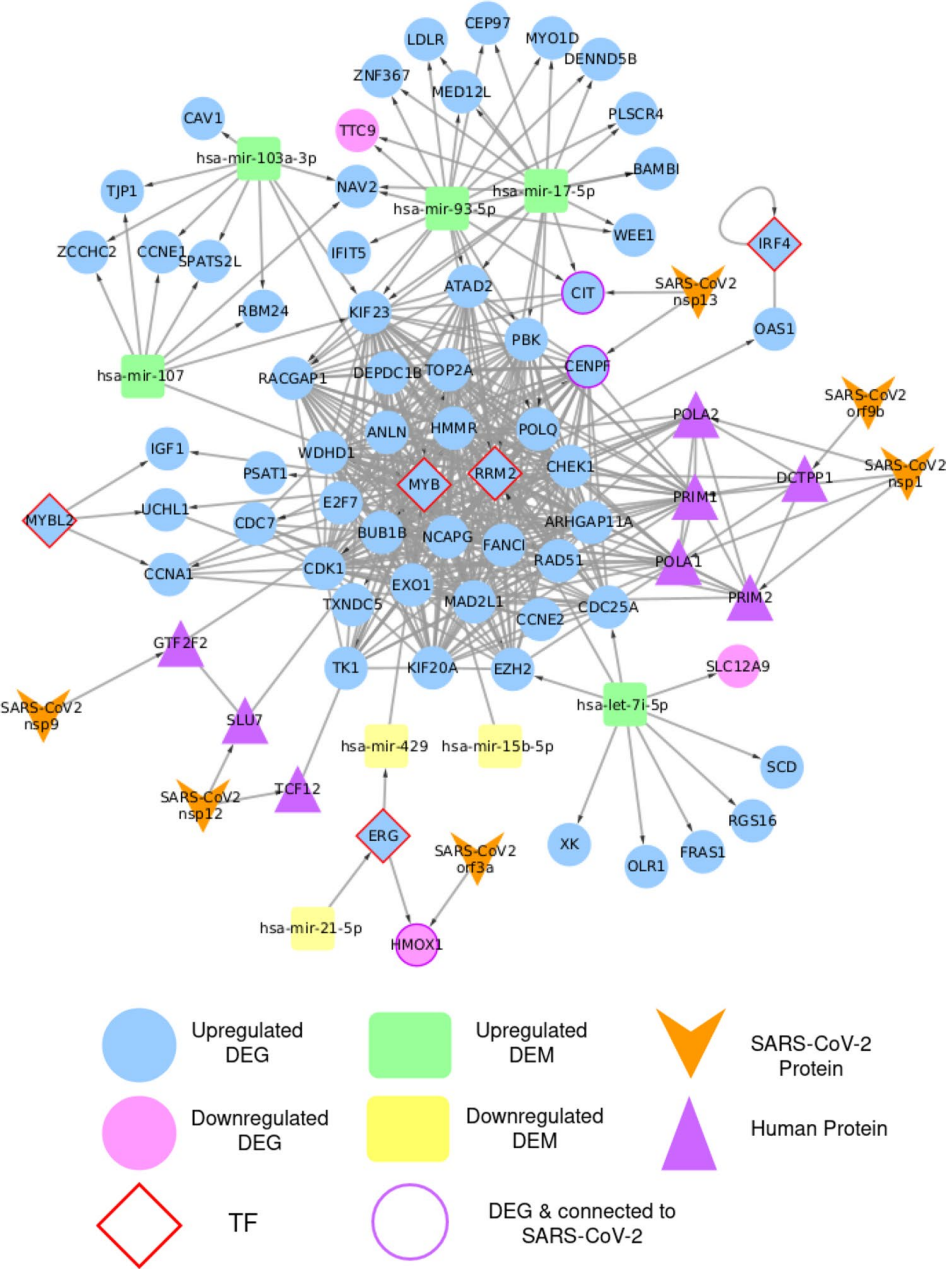
Core network consists of 86 nodes and 457 edges. It contains TFs, potential biomarkers, and their first neighbors. It also includes viral proteins connected to any of the selected nodes. Node’s color and shape are mapped to node type as labeled in the figure. Border is colored to highlight special cases of nodes. Directed edges represent regulatory and virus-host interactions while undirected edges represent human PPIs.

To narrow down the All in One network and explore the influential nodes, we defined the Core network that consists of 86 nodes and 457 edges. It includes 5 TFs, the 5 previously identified potential biomarkers, and their 70 directly connected nodes. Additionally, we included 6 viral proteins that interact with any of the previously selected nodes as shown in Fig. 5. The network nodes were ranked for more investigation based on the Maximal Clique Centrality (MCC) method (Supplementary Table 6). We found that the first 21 genes have the highest MCC score of 9.22E+13 (Table 2). Twenty of them are upregulated DEGs such as CDK1, HMMR, EXO1, BUB1B, and TOP2A connected to TFs (MYB and RRM2), CENPF (connected to nsp13). PBK is also connected to the previously mentioned nodes and two upregulated miRNAs (hsa-mir-93-5p and hsa-mir-17-5p). Table 2 also listed PRIM1 as a top ranked gene but it does not have $Log_2FC$ and padj values as it was retrieved from the SARS-CoV-2-human protein-protein interactions network in Gordon et al. study^24^. It is connected to SARS-CoV-2 nsp1, 17 upregulated DEGs such as CDK1, TOP2A, BUB1B, CENPF, and RRM2-TF.

**Table 2:** Top ranked nodes of the Core network based on MCC algorithm.

Node	$Log_2FC$	padj	MCC
TOP2A	3.032766241	1.23E−23	9.22E+13
PBK	3.286974214	1.87E−19	9.22E+13
RRM2	2.563244695	7.40E−19	9.22E+13
BUB1B	2.848843114	9.48E−18	9.22E+13
EXO1	2.569288856	1.65E−15	9.22E+13
HMMR	2.671877231	4.34E−14	9.22E+13
NCAPG	2.319663731	3.83E−14	9.22E+13
CENPF	2.207653	7.83E−13	9.22E+13
CDK1	2.444515359	1.88E−12	9.22E+13
CHEK1	1.63245883	2.46E−09	9.22E+13
RAD51	1.970628548	9.30E−09	9.22E+13
KIF20A	2.410368374	1.81E−09	9.22E+13
ANLN	2.372484524	2.12E−08	9.22E+13
ATAD2	1.296402031	3.01E−06	9.22E+13
WDHD1	1.334236601	2.11E−06	9.22E+13
KIF23	1.315400626	6.74E−05	9.22E+13
ARHGAP11A	1.243130514	3.70E−05	9.22E+13
RACGAP1	0.994416067	0.000535663	9.22E+13
FANCI	1.133952503	0.000318433	9.22E+13
MAD2L1	1.157533489	0.003931285	9.22E+13
PRIM1	N/A	N/A	9.22E+13

### Screening of FDA-approved drugs with probable potential for COVID-19 treatment

**Figure 6:**
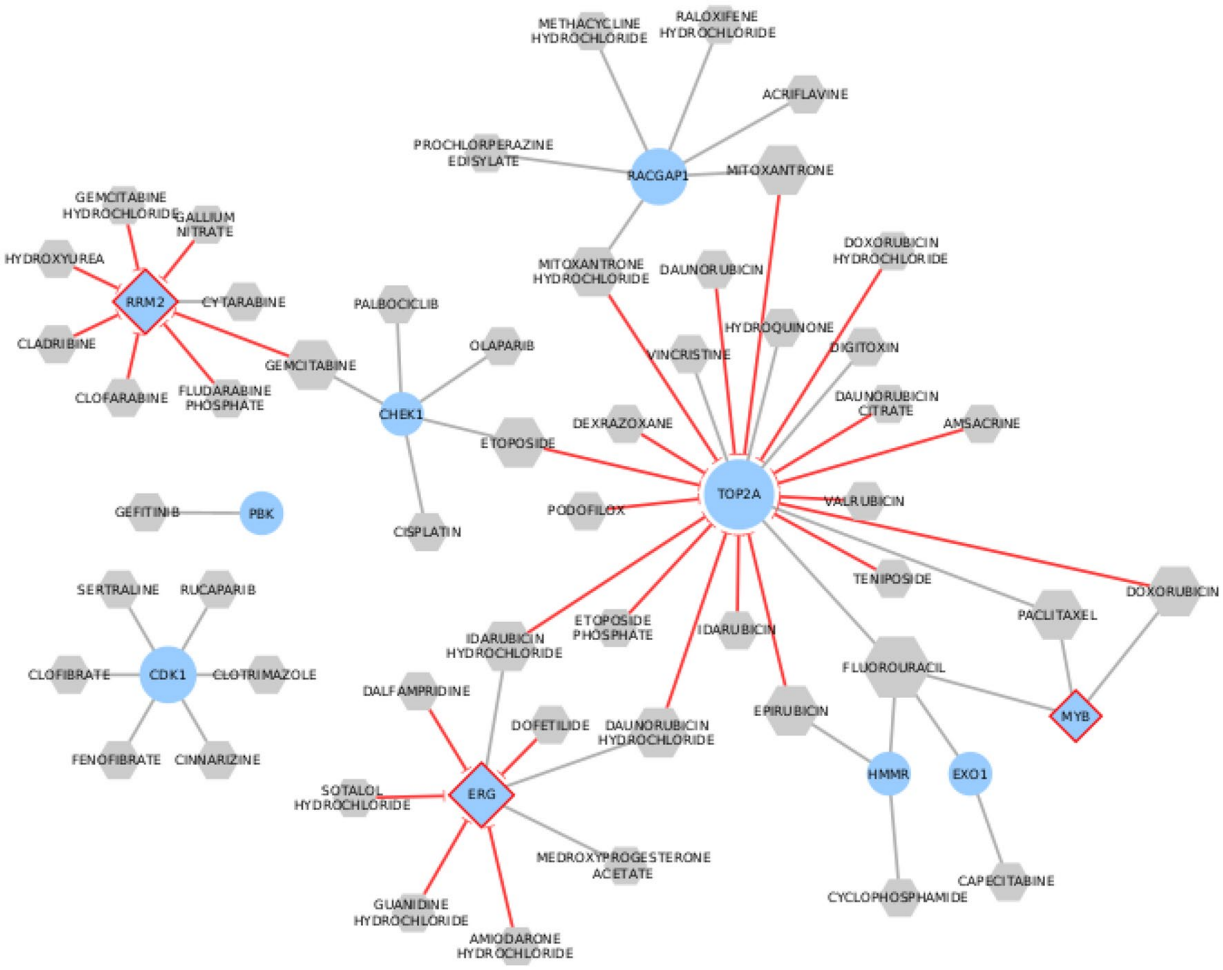
Drug-gene network consists of 62 nodes connected by 64 edges. It contains 7 upregulated DEGs (cyan ellipses), 3 TFs (cyan diamonds with red border), and 52 approved drugs (gray hexagons). Red edges with T shape arrows represent inhibitor or blocker interaction types while gray edges have missing interaction type. Top five targeted genes are represented by large nodes as well as the multi-target drugs.

Drug Gene Interaction Database (DGIdb) web resource^43^ was used to retrieve FDA-approved drugs that interact with 25 genes including the top ranked nodes of the core network (Table 2) and the identified TFs. We found 10 out of the 25 genes (7 upregulated DEGs and 3 TFs) have 64 interactions with 52 approved drugs (Fig. 6 and Supplementary Table 7). TOP2A has the highest number of inhibitory interactions among all genes with 17 drugs such as etoposide, exrazoxane, valrubicin, teniposide, and amsacrine. RRM2-TF can be inhibited by 7 drugs such as gallium nitrate and cladribine, while 5 drugs can block ERG-TF such as sotalol hydrochloride and amiodarone hydrochloride. We also found 10 drugs that could be potential multi-target drugs, such as fluorouracil that targets four genes (HMMR, EXO1, TOP2A, and MYB-TF). Additionally, each of the four anthracycline antitumor antiobiotics (doxorubicin, daunorubicin, idarubicin, and epirubicin) can target two genes.

### Validation of hub genes and core DEMs

**Figure 7:**
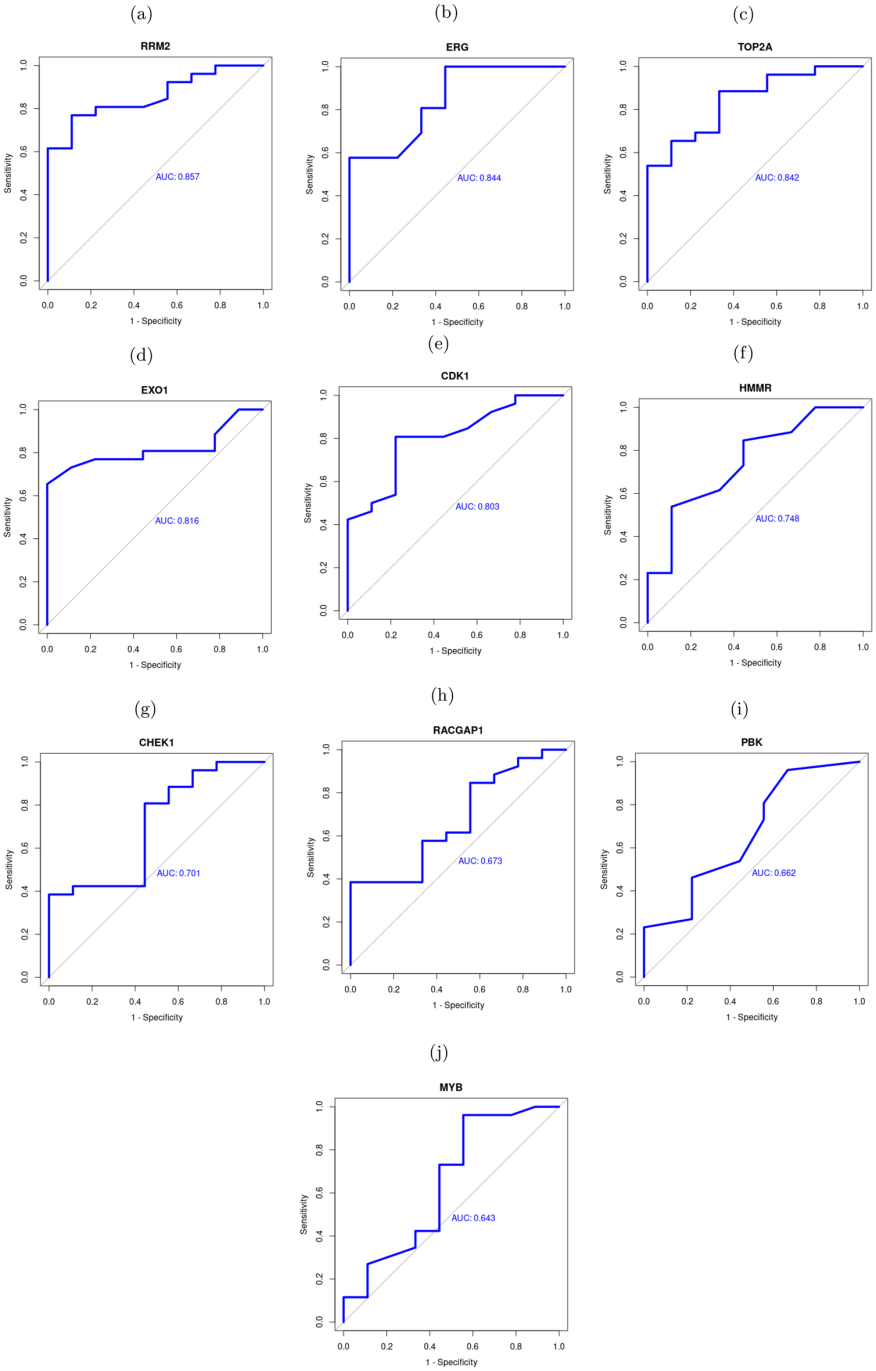
ROC curves of 10 hub genes between COVID-19 patients and healthy controls.

**Figure 8:**
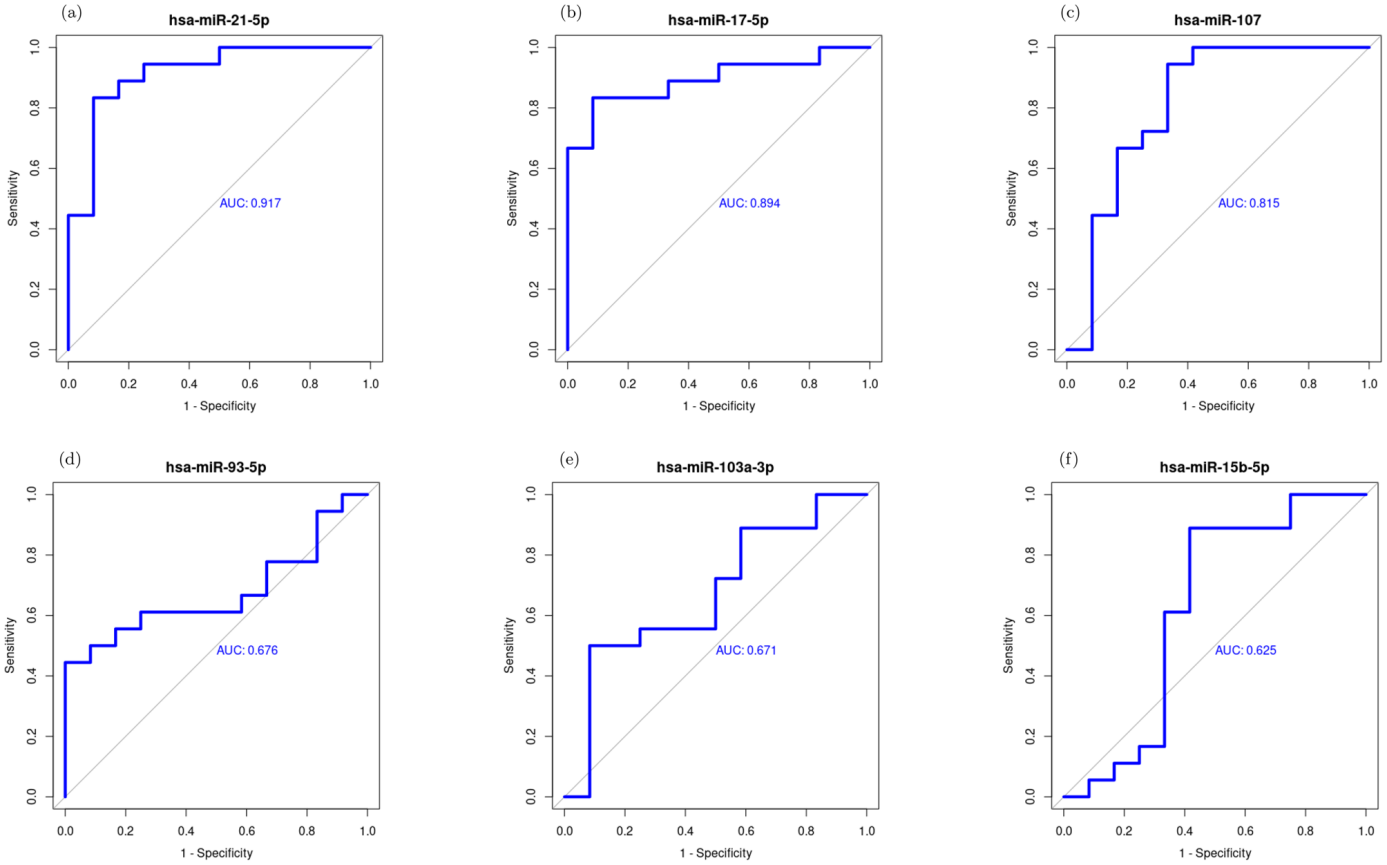
ROC curves of the identified DEMs of the Core network between COVID-19 patients and healthy controls.

Based on mRNA expression profiles of COVID-19 patients and healthy controls of GSE196822, we evaluated the top 10 ranked genes from the core network (Table 2) and also were identified at the previous section as drug targets (Fig. 6). Five hub genes showed high potential to discriminate COVDI-19 patients group from the controls (RRM2, ERG, TOP2A, EXO1, and CDK1) with area under the curve (AUC) values > 0.8 as shown in Fig. 7. AUC values of the other five genes ranged from 0.64 to 0.75. Similarly, the diagnostic potential of the eight identified DEMs of the Core network (Fig. 5) was determined based on miRNA profiles of COVID-19 vs. healthy controls (GSE176498^79^). AUC values showed that the upregulated DEMs (hsa-mir-17-5, hsa-mir-107, hsa-mir-93-5p, and hsa-mir-103a-3p) may be considered as candidate biomarkers for SARS-CoV-2, especially the first two miRNAs with AUC values of 0.89 and 0.82 (Fig. 8). Additionally, the downregulation of hsa-mir-21-5p is significantly associated with SARS-CoV-2 infection with AUC value of 0.92 (Fig. 8).

## Discussion

Effective diagnostic and therapeutic strategies against viruses rely heavily on comprehending the mutual interplay between the virus and its host. Transcriptomic and network analyses are the key players in understanding those interactions, thus SARS-CoV2-host interactions are currently under research^25^. We conducted an in silico analysis of DEGs, DEMs, and human PPIs to infer enriched pathways and potential drug targets in patients and lung cell lines infected with SARS-CoV-2. Although we expect the immune pathways to be the most significantly enriched in the infected samples, to our surprise, pathways involved in regulating the cell cycle progression and DNA replication and repair were ranked the top Reactome terms. As viruses lack the cellular machinery required for genome replication and proteins synthesis, they hijack host cell machinery at the site of infection. Viruses either promote or arrest cell cycle progression for their benefit^26^. Hence, viruses may promote host cell cycle arrest at the active metabolic state to utilize all the cellular resources of nucleotides and proteins and produce new viral cells^27^. In SARS-CoV-2 infected cell lines, cell cycle arrest patterns were observed^28^. However, in peripheral blood samples in our study as shown in Table 1, host cell cycle progression was induced . This is further supported by other studies on peripheral blood samples of infected patients in which cell cycle progression was the highest signal among other cellular, metabolic, and signaling pathways^29–31^. This may be attributed to the fact that virally infected B cells signal cell cycle progression^32^. Intriguingly, cell cycle progression pathways are also enriched in SARS-CoV-2-infected human gastrointestinal cells^33^. In addition to our study, the aforementioned studies confirm that the impact of SARS-CoV-2 on host cell cycle regulation is yet not fully understood^32^ and requires further in vivo testing and validation.

miRNAs are key players in gene expression regulation through interaction with genes, transcription factors, and other regulatory miRNAs. Additionally, they serve as biomarkers for the diagnosis of various diseases^34^. In our study, hsa-mir-93-5p and hsa-mir-17-5p were found to be significantly upregulated. In a study on DEGs in peripheral blood samples of patients with SARS-CoV-2, these two miRNAs were deduced as potentially interacting miRNAs^30^. hsa-mir-93-5p was deduced as a signature that may distinguish patients with COVID-19 from healthy individuals^35^. These findings may nominate both miRNAs as candidate biomarkers for the viral infection using only blood samples from the patients. On the other hand, hsa-mir-21-5p, hsa-mir-10a-5p and hsa-mir-15b-5p were significantly downregulated in our analysis with AUC values of 0.9, 0.85, and 0.6, respectively. On a study on patients with SARS-CoV-2 infection, hsa-mir-21-5p were found to be downregulated in peripheral blood samples of both moderate and severe infections and correlated with immune hyperactivation^36^. hsa-mir-10a-5p is involved in regulating host immune responses. For protection from host immune attacks, SARS-CoV-2 suppresses human hsa-mir-10a-5p and dysregulates its transcription^15^. hsa-mir-15b-5p was downregulated in our analysis and it was experimentally proven to bind to RNA-dependent RNA polymerase structure of SARS-CoV-2 leading to inhibition of viral replication^40^. Through targeting this gene, hsa-mir-15b-5p acts as a natural antiviral agent and thus it may serve as a miRNA-based treatment for SARS-CoV-2^39^. Both hsa-mir-107 and hsa-mir-103a-3p with AUC of 0.815 and 0.671, respectively were predicted to be bound via three binding sites to SARS-CoV-2 with perfect matching to the seed region^37^. hsa-mir-103a-3p specifically binds to the viral nucleocapsid protein coding region^38^.

Transcription factors are regulators that govern the gene expression process in living organisms. Through binding to specific sites on the DNA, direct interaction with miRNAs, or both mechanisms, TFs play a vital role in cell differentiation and even human diseases^41^. Based on TFmiR results, five TFs (MYB, RRM2, ERG, MYBL2, and IRF4) were highlighted in the RNA regulatory network (Fig. 4). MYB is especially interesting because it was found to regulate 17 genes (TOP2A, CCNA1, CENPF, CDK1, BUB1B, CDC7, MAD2L1, RAD51, TK1, TXNDC5, PSAT1, HMMR, EXO1, UCHL1, TXNDC5, EZH2, WDHD1, IGF1) and be regulated by miRNAs (hsa-mir-107, hsa-mir-15b-5p, hsa-mir-103a-3p). Hence, we propose MYB as a golden drug target for COVID-19 treatment. In a study on transcription regulation in leukocytes of patients infected with SARS-CoV-2, MYB regulation mechanisms were upregulated in patients with poor prognosis and related to the severity of the disease^42^. Since many of the genes regulated by MYB are involved in the cell cycle, anticancer drugs might serve as potential repurposed drugs for COVID-19. According to DGIdb, drugs such as doxorubicin, paclitaxel and fluorouracil have an inhibitory effect on MYB^43^. RRM2 (AUC=0.857) is one of the principal genes responsible for the de novo synthesis of nucleotides during DNA replication. In addition to its role as a tumor promoter in various cancers, RRM2 was found to be actively interacting with proteins of RNA viruses such as mouse hepatitis virus and hepatitis C virus^44,45^. The RRM2-viral proteins interactions further boost viral replication^45^. Moreover, the E7 protein of the human papillomavirus induces the overexpression of RRM2; thus, it promotes human cervical cancer^46^. Therefore, RRM2 is a promising target for repurposed anti-cancers as it was found to be overexpressed in our study and probably interact with SARS-CoV-2 proteins. Gemcitabine, cytarabine, and hydroxyurea are among the FDA-approved anti-cancer drugs that target RRM2 and are suggested by the drug-gene network. ERG (AUC=0.844) is a transcription factor that plays a role in embryo development. It is also an oncogene that contributes to leukemia and Ewing’s sarcoma. Lately, ERG has been associated with prostate cancer through gene fusion to TMPRSS2^47^. TMPRSS2 has gained particular interest since the beginning of the pandemic as it facilitates the entry of SARS-CoV-2 into host cells and viral activation. Ergo, patients with prostate cancer might experience unusual pathogenesis of COVID-19^48^. In our analysis, ERG was overexpressed and notably interacting with multiple genes. It can be targeted by many drugs, such as the two FDA-approved antiarrhythmic drugs, sotalol, and amiodarone.

Coronaviruses proteins might directly interact with the host cell proteins involved in the cell cycle to recruit the necessary machinery for viral replication^27^. CENPF and CIT are two proteins involved in the stages of cell cycle progression. In our analysis as illustrated in Fig. 5, they were differentially expressed and bound to nsp13 of SARS-CoV-2. Nsp13 helicase is highly conserved in the coronaviruses family and pivotal in viral replication. Further studies are needed to investigate the effect of targeting CENPF and CIT as a possible therapeutic strategy to treat COVID-19. It is worth mentioning that the overexpression of CENPF and CIT, as is the case in this study, has been associated with different types of cancer such as cervical cancer, hepatocellular carcinoma, osteosarcoma, bladder cancer, and esophageal squamous cell carcinoma^49–53^. Targeting MYB may be also an alternative strategy to inhibit CENPF and indirectly control viral replication. Another SARS-CoV-2 protein found to bind to HMOX1 host protein is ORF3a. ORF3a is a transmembrane protein that aids in translocating the replicated virions outside the infected cells. ORF3a might also induce cellular apoptosis, and it is involved in the host immune response to the virus^54,55^. HMOX1 encodes for heme oxygenase protein (HO-1) that catabolizes human heme. Lungs are one of the main sites of HO-1 expression for protective purposes. In viral infections, the expression of HO-1 decreases, which is further supported by our study as a differentually expressed downregulated protein. HMOX1 expression might be a prognostic biomarker for COVID-19 disease^56^. In addition, the HMOX1 pathway regulates platelets aggregation and poses anti-inflammatory effects. As a defense mechanism, ORF3a binds to HMOX1 to inhibit mechanisms hindering viral replication. Thus, drugs targeting HMOX1-ORF3a binding or enhancing expression of HMOX1 gene can be suggested as new alternatives for COVID-19 treatment. A study proposed the use of the leaf extract of the neem plant to overexpress HMOX1 gene and target SARS-CoV-2 infection^57^.

To conclude the potential repurposed drugs for SARS-CoV-2, we constructed a drug-gene network (Fig. 6) based on the potential drug targets identified in the Core network (Fig. 5). Since the cell cycle progression pathways were significantly enriched in our study, most of the suggested drugs are anticancer drugs that target genes involved in the cell cycle. Multiple studies also supported the repurposing of anticancer drugs to treat COVID-19^26,30,58–63^. This can be explained by the similar influence that pathogens and cancers have on the biologically active biological processes in the human body^26^.

Using DGIdb, all sources of drug-gene interactions were included to ensure a comprehensive thorough search. Among the 25 top ranked genes and TFs, only 10 genes showed interactions with 52 FDA-approved drugs: CDK1, HMMR, EXO1, PBK, RRM2, CHEK1, RACGAP1, MYB, ERG, and TOP2A. TOP2A (AUC = 0.842) is a crucial gene in the DNA replication process during the cell cycle. It was identified as a central hub gene in a study on comparing the gene expression profiles of blood samples from patients with COVID-19 with healthy controls^64^. According to the drug-gene network constructed, it was inhibited by different classes of anti-cancers such as anthracyclines, mitotic inhibitors, anti-tumor antibiotics, and topoisomerase II inhibitors. Etoposide is a topoisomerase II inhibitor that is currently under phase II clinical trial for COVID-19 therapy^65^. Moreover, hydroxyquinone and digitoxin also have a potential inhibitory effect on TOP2A. CDK1 (AUC=0.803) is a cyclin-dependent kinase that modulates cell cycle progression. Several viruses can control the expression of CDKs to create a suitable cellular environment for viral replication. CDK inhibitors have been proposed to limit viral infectivity to the cells in general, and SARS-CoV-2 in particular^62^. CDK1 was an overexpressed hub gene in our study and previous studies^30^. Subsequently, CDK1 inhibition may offer a therapeutic strategy to fight COVID-19. According to our drug-gene network, this can be achieved by repurposing riviciclib, dinaciclib, milciclib, and others.

Drugs targeting multiple genes are of particular interest within the drug-gene network. This can be explained by the fact that these repurposed drugs, when given as single agents, would be more efficient and have fewer side effects than drug combinations. For example, in our study, fluorouracil targets four genes with a transcription factor included. Carmofur, a fluorouracil-based anti-cancer, and doxorubicin have been proposed for COVID-19 treatment^30,65^. Despite being promising drug targets, more studies need to be conducted to validate the use of repurposed anti-cancer drugs to fight COVID-19 with the proper dosing. This is due to the detrimental side effects of anti-cancers on normal cells and the quality of life in general.

## Conclusion

We sought potential drug targets, biomarkers, and repurposed FDA-approved drugs to help early diagnosis and treatment of COVID-19. Our integrative bioinformatics analyses identified MYB, RRM2, ERG, CENPF, CIT, TOP2A, and HMOX1 as candidate drug targets. Both hsa-mir-93-5p and hsa-mir-17-5p were identified as candidate biomarkers for COVID-19 infection. In addition, hsa-mir-15b-5p is a promising miRNA-based antiviral agent. The key drugs retrieved were members of anthracyclines, mitotic inhibitors, anti-tumor antibiotics, and CDK1 inhibitors. Additionally, hydroxyquinone and digitoxin are potent TOP2A inhibitors. The analysis enforced the repositioning of fluorouracil and doxorubicin, especially those with multiple drug targets assuming less probability of resistance. Our study provides promising results that could facilitate developing diagnostic and therapeutic approaches against the COVID-19 pandemic.

## Methods

### Data retrieval

Two gene expression profiling datasets were retrieved from the Gene Expression Omnibus (GEO) repository. The first RNA-seq data (Accession no. GSE157103)^66^ was generated on Illumina NovaSeq 6000 platform and consisted of 126 blood samples; 100 COVID-19 patients and 26 non-COVID-19 samples. The dataset included additional metadata about the samples, such as age, gender, and clinical data. It had 74 males and 52 females. The age range was from 21 to 89 years. PCA has been utilized to ensure that factors such as age, gender, or Charlson severity index score do not affect gene expression. The second dataset (Accession no. GSE148729)^67^ was generated on Illumina NextSeq 500 platform. It consisted of different gene expression profiling measurements using bulk and single-cell sequencing. We only retrieved read counts of miRNAs from mock and infected epithelial lung cancer cell lines (Calu-3) 24 h post-infection (two replicates for each group) for the expression analysis.

### Gene expression and pathway enrichment analyses

Differential expression analysis (DEA) was performed to identify the differentially expressed genes (DEGs) in the COVID-19 patients and the differentially expressed miRNAs (DEMs) in the SARS-CoV-2 infected human cell lines, respectively. In both cases, DESeq2 Bioconductor package^68^ was used to run the analysis. Messenger RNAs (mRNAs) and miRNAs with adjusted *P*-value < 0.05 and $|Log_2FC|$> 0.5 were considered as DEGs and DEMs, respectively. To reveal biological insight into the resulting DEGs and their association with the virus infection, pathway enrichment analysis was performed using Reactome analysis tool^69^. False discovery rate (FDR) cutoff of 0.01 was used to highlight the significantly enriched pathways.

### Network construction and analysis

Multiple networks were constructed for an integrated analysis of the identified DEGs and DEMs as follows:

1. An RNA regulatory network between the identified DEGs and DEMs was created using the TFmiR web server^70^. TFmiR highlights transcription factors (TFs) if they exist in the given input of DEGs and provides their interactions with the given miRNAs and genes in the network.
2. For SARS-CoV-2-human protein-protein interactions (PPIs) retrieval, we used Gordon et al. study^24^. Using affinity-purification mass spectrometry, the authors reported the human proteins that were physically associated with SARS-CoV-2 expressed proteins in human cells. The dataset is publicly available at STRING v11.5 database^71^. It contains 332 human proteins connected to 27 SARS-CoV-2 proteins.
3. STRING v11.5 database was used to retrieve PPIs of the identified DEGs, the human proteins identified in the second network, and between both categories. Co-expression, experimental validation, and information from databases were the only interaction evidences included in the network with a minimum confidence score of 0.4.

All the aforementioned different types of interactions were integrated into one complete network, referred to as the “All in One network” using Cytoscape v3.9.0 software^72^. It has also been utilized in visualization and analysis. The “Core network” was extracted from the All in One network to understand the complex interactions better. It includes TFs, upregulated DEMs identified from the RNA regulatory network, and their first neighboring nodes. We also added the interacting viral proteins with any of the mentioned nodes. Then, Maximal Clique Centrality (MCC) algorithm was employed to identify and prioritize the hub genes for further investigation using Cytoscape plugin cytoHubba^73^. Among the 11 topological analysis methods provided by cytoHubba, we selected MCC for ranking as it showed more precise performance over other centrality measures such as degree, closeness, and betweenness^73^.

### Drug repurposing

Drug repurposing is considered an effective alternative strategy for the emerging pandemic. Accordingly, we chose the Drug Gene Interaction Database (DGIdb) v4.2.0^43^ to suggest drugs that target both the top ranked genes of the Core network and the previously identified TFs. DGIdb is an expert-curated database that integrates 100, 273 drug-gene interactions from 22 source databases such as DrugBank^74^, Drug Target Commons^75^, PharmGKB^76^, and Chembl^77^. It has more than 40, 000 genes and 10, 000 drugs from different drug classes. To filter the interacting drugs with the query genes, we chose only approved drugs filter to make sure we included all approved drug classes and not only antineoplastics or immunotherapies. We also included all drug interactions databases, all gene categories, and all drug-gene interaction types.

### ROC curve analysis

The receiver operating characteristic (ROC) curve analysis was performed to assess the ability of the identified hub genes and DEMs of the Core network to distinguish between COVID-19 patients and healthy controls groups. pROC R package^78^ was used to generate ROC curves and calculate the area under the curve (AUC) values for the hub genes and DEMs from GEO datasets GSE196822 and GSE176498^79^, respectively.

## Supplementary Information

Supplementary Information 1.

## Data Availability

The read counts of gene expression profiling datasets used for differential expression analysis and ROC analysis are available in the GEO repository. GSE157103 for Overmyer et al.^66^, GSE148729 for Wyler et al.^67^, GSE196822 for Banerjee U et al., and GSE176498 for Gutmann C et al.^79^. All results generated by the study analyses are included in this published article (and its Supplementary Information file).

## Code availability

The code for reproducing the transcriptomic analysis results will be made available at the Github repository See Code https://github.com/ComputationalBiologyLab/Transcriptomic-analysis-of-SARS-CoV-2.
